# Serratia marcescens as an Uncommon Cause of Infection Following Craniectomy: A Case Report and Literature Review

**DOI:** 10.7759/cureus.86673

**Published:** 2025-06-24

**Authors:** Shreya Veggalam, Venkataramana Kandi

**Affiliations:** 1 Medicine, Prathima Institute of Medical Sciences, Karimnagar, IND; 2 Clinical Microbiology, Prathima Institute of Medical Sciences, Karimnagar, IND

**Keywords:** abdominal wall, craniectomy, neurosurgery, postoperative, serratia marcescens, wound infections

## Abstract

*Serratia marcescens* (*S*. *marcescens*) is a common bacterial species isolated from patients’ specimens. Medical equipment like catheters, cannulas, tubing, dressing materials, and surgical instruments can become colonized by *S*. *marcescens*. Critically sick patients are the main victims, especially those in intensive care units or surgical settings where extended hospital stays and the use of broad-spectrum antibiotics are typical. Although *S*. *marcescens* is commonly linked to bloodstream infections, respiratory tract infections, and urinary tract infections, it is comparatively uncommon to be involved in wound infections following surgery. We describe a rare instance of a soft tissue infection brought on by *S*. *marcescens* at the location of the abdominal wall where autologous bone flaps were temporarily stored after a decompressive craniectomy. The patient had symptoms of a localized infection, which led to further evaluation and targeted therapy. Culture and antibiotic sensitivity tests guided the selection of drug therapy. This case demonstrates the unusual way that *S*. *marcescens* manifests in soft tissue infections following neurosurgery, emphasizing how crucial it is to follow strict aseptic procedures, particularly when working with surgical bone flaps. Knowledge of these atypical organisms is crucial for timely diagnosis and effective treatment, especially in critical care and postoperative settings.

## Introduction

*Serratia marcescens* (*S. marcescens*) is a Gram-negative bacterium and is a member of the *Enterobacteriaceae* family. Because of its highly identifiable red-colored colonies and low virulence, this saprophytic bacterium is frequently utilized as a biological marker to detect water contamination. Depending on the colonies' age and the type of culture medium, *Serratia* can produce a non-diffusible and insoluble pigment called prodigiosin, which can have a color ranging from dark red to pink. *Serratia* is a genus consisting of aerobic and motile bacilli that includes 23 species, six of which include *S. marcescens*, *S. plymuthica*, *S. liquefaciens*, *S. rubidaea*, *S. odorifera*, and *S. fonticola*, have been linked to human illnesses. *S. marcescens* is most frequently isolated from human clinical specimens [1,2].

Over the past three decades, *S*. *marcescens *has significantly contributed to hospital-acquired infections or healthcare-associated infections (HAIs), confirming it as an opportunistic pathogen [3]. In addition to causing severe bacteremia in hospitalized patients, *Serratia-*associated bacteremia raises the mortality rate for patients compared to those with other pathogen-caused bacteremia [4]. The misuse of antibiotics in intensive care units (ICUs) and inpatients before and after surgery has led to an increase in multidrug-resistant (MDR) *Serratia *infections. Among the numerous infectious diseases that *S*. *marcescens *can cause are peritonitis, wound infections, urinary, respiratory, and biliary tract infections, as well as potentially lethal infections associated with intravenous catheters. *S*. *marcescens *has been transforming to develop antimicrobial resistance (AMR) due to the use of preventive antibiotics in hospital ICUs [5].

A frequent complication after surgery, postoperative wound infections have a complicated and multifaceted pathogenesis. They are the main cause of HAIs in surgical patients and are also referred to as surgical site infections (SSIs). A craniectomy is a surgical procedure in which the skull bone flaps/cranial vault are removed to relieve pressure on the brain, usually caused by bleeding or swelling following trauma or stroke. The patient's skull bone flaps may be temporarily preserved subcutaneously in the abdominal wall for future reconstruction rather than being replaced right away. As a result, patients are subjected to a second surgical incision that is distinct from the site of craniectomy. We report a case of abdominal wall abscess at the skull bone flap storage site caused by *S. marcescens* following decompressive craniectomy in a stroke patient.

## Case presentation

A 63-year-old man was brought to the hospital with complaints of a painful lump on the right side of his abdomen and fever in August 2019. The patient revealed that the swelling began slowly and increased in size over three weeks. Although there was no diurnal variation, the patient claimed to have had episodes of fever and chills. The patient has neither diabetes nor hypertension, as revealed by normal blood glucose levels and blood pressure. Further, there was no evidence of blood-borne viral infections. Clinical history revealed that the patient had undergone an emergency decompressive craniectomy 21 days prior due to an acute ischemic stroke that affected the right frontal, temporal, and parietal (FTP) areas of the brain. After the cerebral edema had subsided, the cranial vault (skull bone flaps) was subcutaneously deposited in the right lumbar region of the anterior abdominal wall as a normal procedure for further cranioplasty.

When the abdomen was examined locally, the enlargement, which measured about 4X1.7 centimeters, was found in the right lumbar area of the anterior abdominal wall. It was oval in shape and horizontal, with a central discharging sinus that exuded purulent material and reddish discoloration of skin surrounding the lesion. There were no aggravating or alleviating variables, and the swelling was accompanied by dull, throbbing discomfort that was non-radiating and unaffected by postural adjustments. Upon palpation, the enlargement was soft, movable, and transilluminate, and the temperature had increased locally.

An abdominal wall abscess at the cranial vault storage site was diagnosed based on clinical symptoms. Under local anesthesia, the patient had an elective incision and drainage. An incision was made along the prior surgical scar site, and the abdominal areas were cleaned and draped under rigorous aseptic conditions. After draining the purulent material and retrieving and properly storing the cranial bone flap, the wound cavity was treated with hydrogen peroxide and povidone-iodine. After achieving hemostasis, the wound was sutured, and close postoperative surveillance was done.

**Figure 1:**
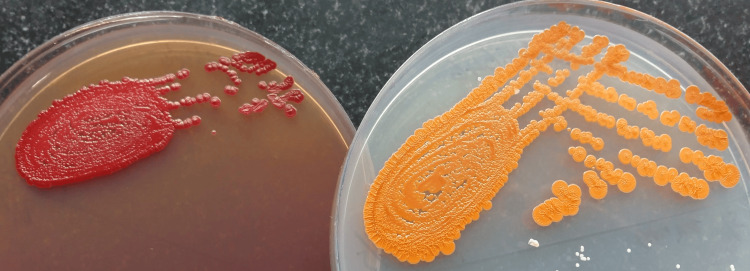
Growth of pigment-producing Serratia marcescens Image credit: Venkataramana Kandi A: growth on MacConkey's agar showing non-diffusible reddish pigment; B: growth on nutrient agar demonstrating non-diffusible orange pigment

The culture of the pus revealed the growth of *S*. *marcescens*, an opportunistic gram-negative bacillus identified by conventional microbiological methods. Growth on MacConkey's agar showed non-diffusible reddish pigment, while growth on nutrient agar demonstrated non-diffusible orange pigment (Figure 1).

**Figure 2:**
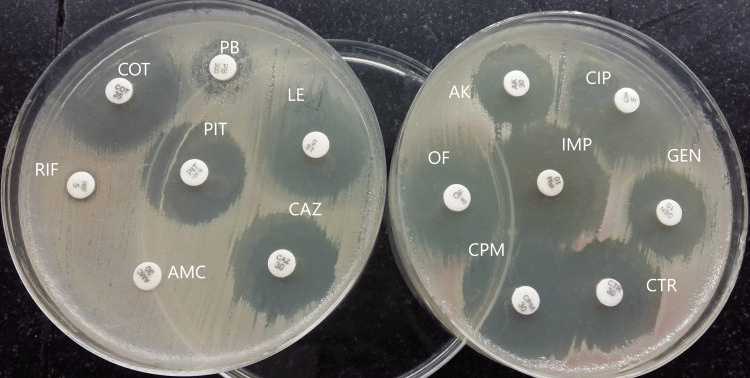
Antibiotic susceptibility testing results of Serratia marcescens Image credit: Venkataramana Kandi RIF: rifampicin; PB: polymyxin B; IMP: imipenem (10 µg); AK: amikacin (30 µg); GEN: gentamicin (10 µg); CIP: ciprofloxacin (5 µg); OF: ofloxacin (5 µg); LEV: levofloxacin (5 µg); COT: cotrimoxazole (1.25/23.75 µg); PTZ: piperacillin-tazobactam (30/6 µg); CAZ: ceftazidime (10 µg); CTR: ceftriaxone (30 µg); CPM: cefepime (30 µg); AMC: amoxycillin-clavulanic acid (20/10 µg)

Antibiotic sensitivity testing (AST) by the Kirby-Bauer disk diffusion method revealed that the bacteria were resistant to rifampicin, polymyxin B, and amoxicillin-clavulanic acid but susceptible to most routinely used antibiotics (Figure 2).

These findings led to the patient being put on targeted antibiotic therapy, which included 1.5 grams of cefoperazone twice a day and intravenous sulbactam for seven days. The supportive treatment consisted of intravenous fluids (normal saline: 80 mL/hour), multivitamins and mineral supplements, paracetamol 650 mg orally twice daily, and 40 mg pantoprazole once daily. The patient responded favorably to the treatment and recovered uneventfully.

## Discussion

Post-operative craniectomy-associated infections remain a very serious complication [6]. In our case, the patient had the cranial vault stored subcutaneously in the right lumbar region of the abdomen and developed a painful abscess. The abdominal abscess formation in a post-operated patient with any abdominal surgery is common, but an infection with *S*. *marcescens *is a rare occurrence. An abdominal abscess consists of cellular debris, enzymes, and liquefied remains from an infection. Therefore, it is prudent to obtain pus after the incision and drainage and send it for culture and AST.

Patients recovering from surgery in an ICU may have a drain at the surgical site, an endotracheal tube, or a urinary catheter indwelling inside their body [5]. *Serratia *can invade a patient's body from the surroundings and lead to several infections like septicemia, meningitis, endocarditis, wound infections, and respiratory and urinary tract infections [7,8].

To prevent postoperative wound infections and other HAIs, which can sometimes be fatal for the patient, antibiotics are typically administered prophylactically both before and after surgery. The goal is to have an adequate concentration of the medication at the wound site to stop microorganisms from getting in and infecting the wound. This is now a significant contributing factor to the increase in HAIs. Treatment becomes challenging when the bacteria develop resistance to the main antibiotic drug subclasses due to the frequent use of antibacterial medications [9,10].

*S*. *marcescens *naturally carries an inducible chromosomal β-lactamase conferring resistance to aminopenicillins, including ampicillin and amoxicillin. Furthermore, *S*. *marcescens *can develop resistance to first- and second-generation cephalosporins but remain sensitive to third- and fourth-generation cephalosporins, monobactams, and carbapenem groups of antibiotics. *S*. *marcescens *can develop AMR quickly, often through mutations in regulatory genes that lead to overproduction of ampicillin (AmpC) β-lactamase or by acquiring resistance genes through plasmids. *Serratia *species can acquire AMR through the combined activity of intrinsic, acquired, and adaptive resistance elements in their genome, which may be driven by the widespread use of antibiotics [11]. AMR has recently been an emerging problem challenging the treatment of infectious diseases. *Serratia *and other bacteria are listed among the newly emerging MDR pathogens due to the extensive development of resistance to cephalosporins, carbapenems, and aminoglycosides [12].

The lack of effective antibiotic treatment and increasing resistance that makes the antibiotics ineffective is showing the potential for *S*. *marcescens *infections to cause increased morbidity and mortality [13]. Studies reveal that *Serratia *has caused nosocomial outbreaks in hospital settings and is a cause of mortality in immunocompromised patients. A combination of bio-typing and random amplified polymorphic deoxyribonucleic acid (RAPD), a type of polymerase chain reaction (PCR), allowed accurate identification of *S*. *marcescens *strains isolated in nosocomial outbreaks at pediatric hospitals; RAPD-PCR helps to analyze the clonal variations in *S*. *marcescens *[14].

The clinical features of skin infections due to *Serratia *include necrotizing fasciitis, infected nodules, cellulitis, ulcers, and abscesses. The cutaneous manifestations are very rare in the infectious spectrum of *S*. *marcescens *[15]. A review of some post-surgery infections caused by *Serratia *demonstrated that extremes of age are a predisposing factor. Type 2 diabetes mellitus (T2DM) was the most common co-morbid condition among infected patients. Abscess, infection of the prosthetic devices, sepsis, meningitis, and post-operative wound infections were commonly associated with *Serratia* (Table 1).

**Table 1:** Demographic and clinical details of infections caused by Serratia marcescens CSF: cerebrospinal fluid; AST: antibiotic susceptibility testing; T2DM: type 2 diabetes mellitus; NA: data not available

Age and sex	Type of sample	Type of post-operative infection	Surgery performed	Treatment and outcome	Any known comorbidities in patients	Reference/citation
63-year-old male	Discharge from the post-operative wound site	Intracranial abscess	Left parietal craniotomy	Antibiotics were prescribed based on AST, and the patient recovered uneventfully	Coronary artery disease with unstable angina	Liu et al., 2024 [16]
72-year-old male	Pus obtained from the fistula at the prepatellar area	Infection of the prosthetic joint area	Total hip arthroplasty	Antibiotics were prescribed based on AST, and the patient recovered uneventfully	T2DM	Karczewski et al., 2023 [17]
1 year 9 months old male baby	Fluid obtained by thoracocentesis	Post-operative infection in a patient after cardiac surgery	Bidirectional Glenn shunt with atrial septectomy	Intravenous antibiotics and the patient recovered uneventfully	NA	Kumaran e t al., 2020 [18]
73-year-old male	Discharge from subconjunctival abscess	Subconjunctival abscess	Ahmed glaucoma implant in a patient with multiple previous ocular surgeries	Antibiotics were initiated after AST results, and the patient recovered uneventfully	T2DM	Sosuan et al., 2020 [19]
5-month-old male baby	Blood	Sepsis	Liver transplantation	Died due to hyperammonemia	NA	Mouat et al., 2018 [20]
54-year-old female	Pus drainage from the infection in the groin region	Cellulitis of the thigh	Trans obturator sling operation	Antibiotics were started, and incision and drainage were performed	NA	Roth et al., 2015 [21]
54-year-old female	Pus obtained from the epidural abscess	Spinal epidural abscess	Decompressive surgery of the spinal cord for hypertrophic vertebral disc	Antibiotics were initiated, and the patient recovered uneventfully	NA	Yang et al., 2014 [22]
56-year-old male	Pus from the infection site	Spondylodiscitis	Anterior cervical discectomy patient	Intravenous antibiotics were initiated, and the patient improved after revision surgery	NA	Kulkarni et al., 2006 [23]
57-year-old male	Blood and CSF	Meningitis	Stapedectomy	NA	NA	Jablokow et al., 1982 [24]

In a recent study conducted in Oman, *S*. *marcescens *(79.4%) was the most commonly identified species*.* This study evaluated antibiotic resistance patterns, risk factors, and disease outcomes related to *Serratia *infections. Adults aged > 60 years (29.4%), infants (28%), and patients receiving critical care unit treatment were the most affected. *Serratia *has proven to be very resistant to beta-lactam antibiotics. They showed high susceptibility rates to imipenem, ciprofloxacin, cotrimoxazole, gentamicin, amikacin, piperacillin-tazobactam, tigecycline, and meropenem. Hemodialysis, mechanical ventilation, pneumonia, and septicemia were the independent risk factors for higher mortality among the participants under study (p < 0.05) [25].

The existing literature confirms the opportunistic nature of *Serratia*. Furthermore, it is concerning that MDR strains are emerging and spreading. To combat AMR and enhance patient outcomes, it is essential to regularly evaluate AST and update clinicians' understanding of antimicrobial susceptibility profiles, antibiotic prescribing strategies, and infection control measures.

## Conclusions

Apart from being present in the environment,* S*. *marcescens *is a common inhabitant in hospital settings. The presence of debilitating diseases like T2DM and immunocompromised conditions, along with a history of surgery, can predispose to *Serratia *infections. The major risk factors for *Serratia *infections include extremes of age (old age and infants) and prolonged hospitalization. Overusing and misusing antibiotics prescribed by physicians can be dangerous, as the organisms are constantly exposed to the same antibiotic and gain resistance against it. Emerging evidence of AMR among *Serratia *and the occurrence of outbreaks indicate the clinical significance of these bacteria. The culture and AST of the organism can drive the physician toward a targeted treatment, improve patient outcomes, and minimize the morbidity and mortality associated with infections.
